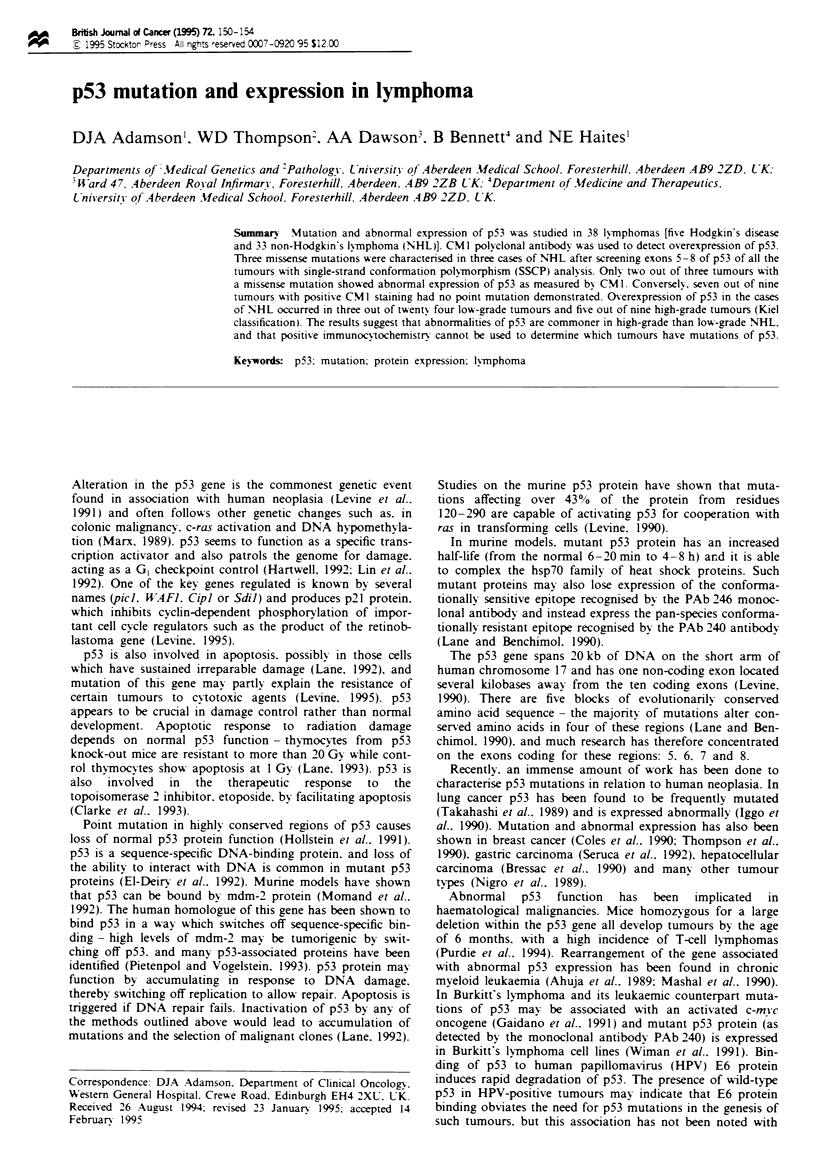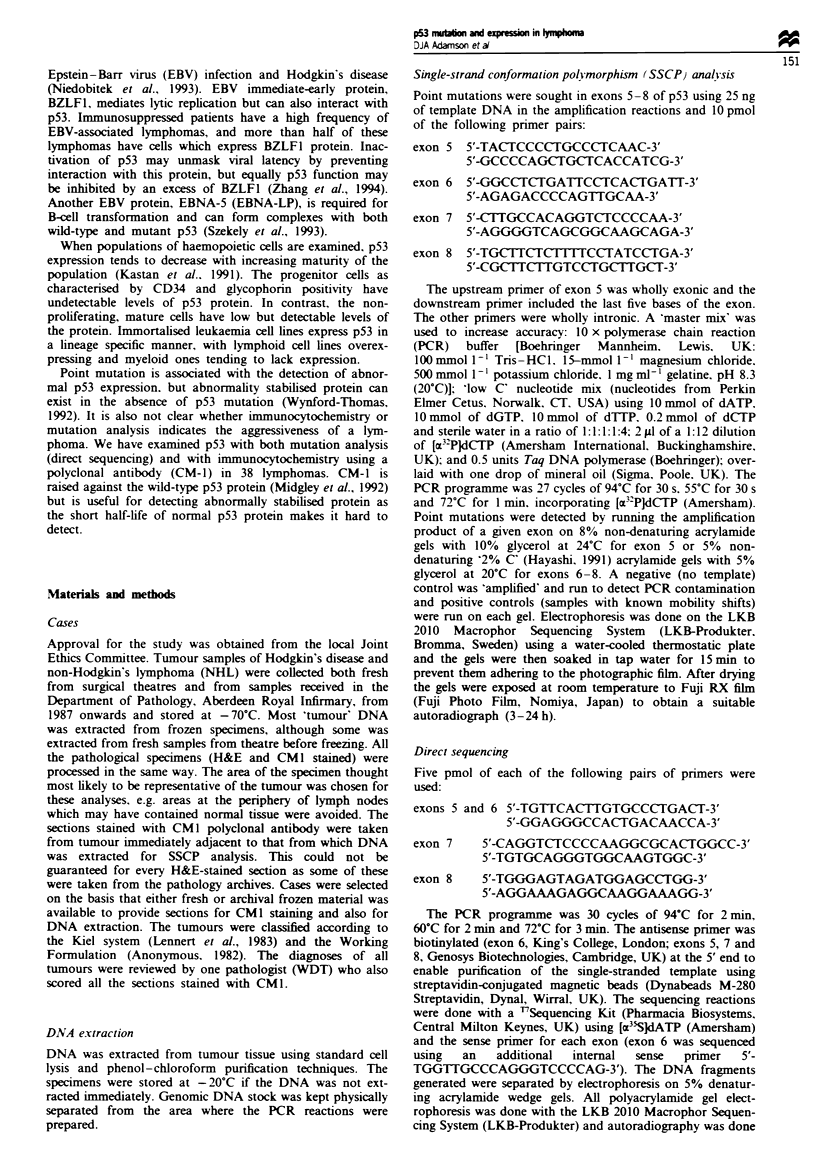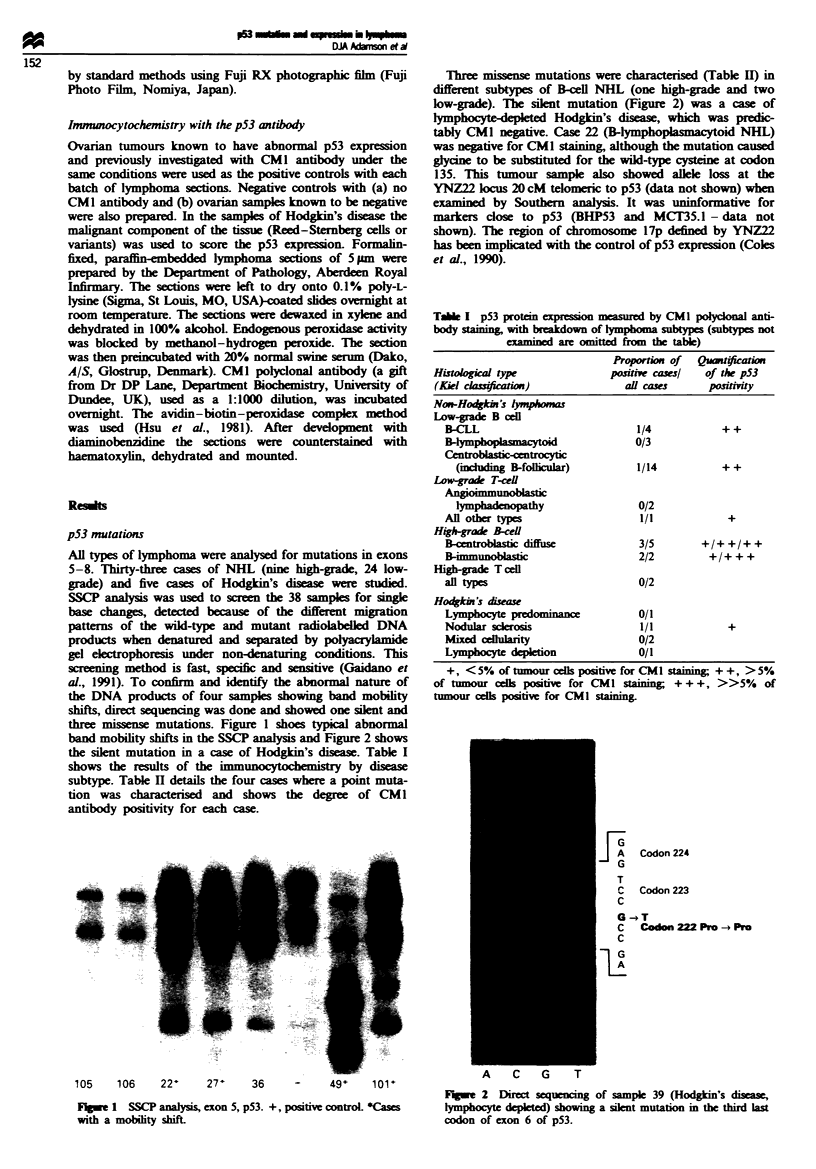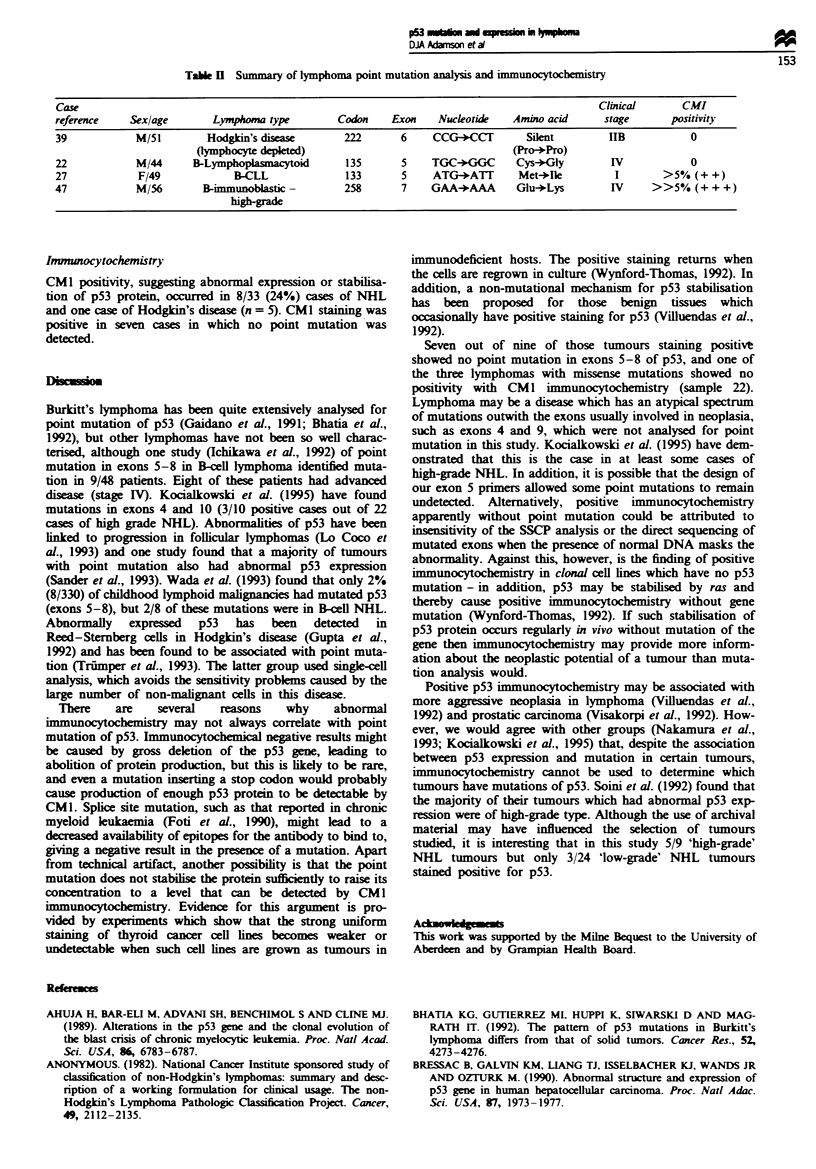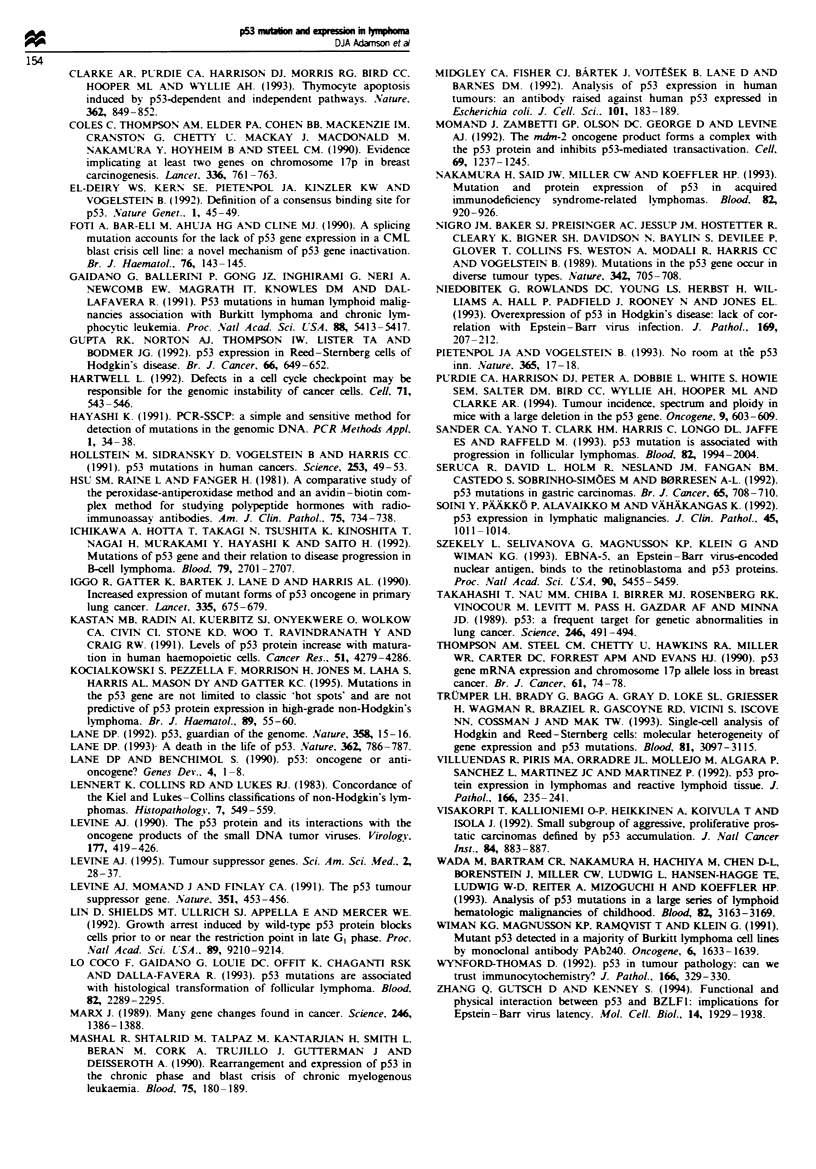# p53 mutation and expression in lymphoma.

**DOI:** 10.1038/bjc.1995.292

**Published:** 1995-07

**Authors:** D. J. Adamson, W. D. Thompson, A. A. Dawson, B. Bennett, N. E. Haites

**Affiliations:** Department of Medical Genetics, University of Aberdeen Medical School, Foresterhill, UK.

## Abstract

**Images:**


© 1995 Stockton Press All rights reserved 0007-0920/95 $12.00

### p53 mutation and expression in lymphoma

DJA Adamson[1], WD Thompson[2], AA Dawson[3], B Bennett[4] and NE Haites[1]

*Departments of [1]Medical Genetics and [2]Pathology, University of Aberdeen Medical School, Foresterhill, Aberdeen AB9 2ZD, UK; [3]Ward 47, Aberdeen Royal Infirmary, Foresterhill, Aberdeen, AB9 2ZB UK; [4]Department of Medicine and Therapeutics, University of Aberdeen Medical School, Foresterhill, Aberdeen AB9 2ZD, UK.*

**Summary** Mutation and abnormal expression of p53 was studied in 38 lymphomas [five Hodgkin's disease and 33 non-Hodgkin's lymphoma (NHL)]. CM1 polyclonal antibody was used to detect overexpression of p53. Three missense mutations were characterised in three cases of NHL after screening exons 5-8 of p53 of all the tumours with single-strand conformation polymorphism (SSCP) analysis. Only two out of three tumours with a missense mutation showed abnormal expression of p53 as measured by CM1. Conversely, seven out of nine tumours with positive CM1 staining had no point mutation demonstrated. Overexpression of p53 in the cases of NHL occurred in three out of twenty four low-grade tumours and five out of nine high-grade tumours (Kiel classification). The results suggest that abnormalities of p53 are commoner in high-grade than low-grade NHL, and that positive immunocytochemistry cannot be used to determine which tumours have mutations of p53.

**Keywords:** p53; mutation; protein expression; lymphoma

Alteration in the p53 gene is the commonest genetic event found in association with human neoplasia (Levine *et al*., 1991) and often follows other genetic changes such as, in colonic malignancy, c-*ras* activation and DNA hypomethylation (Marx, 1989). p53 seems to function as a specific transcription activator and also patrols the genome for damage, acting as a $G_1$ checkpoint control (Hartwell, 1992; Lin *et al*., 1992). One of the key genes regulated is known by several names (*pic1*, *WAF1*, *Cip1* or *Sdi1*) and produces p21 protein, which inhibits cyclin-dependent phosphorylation of important cell cycle regulators such as the product of the retinoblastoma gene (Levine, 1995).

p53 is also involved in apoptosis, possibly in those cells which have sustained irreparable damage (Lane, 1992), and mutation of this gene may partly explain the resistance of certain tumours to cytotoxic agents (Levine, 1995). p53 appears to be crucial in damage control rather than normal development. Apoptotic response to radiation damage depends on normal p53 function - thymocytes from p53 knock-out mice are resistant to more than 20 Gy while control thymocytes show apoptosis at 1 Gy (Lane, 1993). p53 is also involved in the therapeutic response to the topoisomerase 2 inhibitor, etoposide, by facilitating apoptosis (Clarke *et al*., 1993).

Point mutation in highly conserved regions of p53 causes loss of normal p53 protein function (Hollstein *et al*., 1991). p53 is a sequence-specific DNA-binding protein, and loss of the ability to interact with DNA is common in mutant p53 proteins (El-Deiry *et al*., 1992). Murine models have shown that p53 can be bound by mdm-2 protein (Momand *et al*., 1992). The human homologue of this gene has been shown to bind p53 in a way which switches off sequence-specific binding - high levels of mdm-2 may be tumorigenic by switching off p53, and many p53-associated proteins have been identified (Pietenpol and Vogelstein, 1993). p53 protein may function by accumulating in response to DNA damage, thereby switching off replication to allow repair. Apoptosis is triggered if DNA repair fails. Inactivation of p53 by any of the methods outlined above would lead to accumulation of mutations and the selection of malignant clones (Lane, 1992).

Studies on the murine p53 protein have shown that mutations affecting over 43% of the protein from residues 120-290 are capable of activating p53 for cooperation with *ras* in transforming cells (Levine, 1990).

In murine models, mutant p53 protein has an increased half-life (from the normal 6-20 min to 4-8 h) and it is able to complex the hsp70 family of heat shock proteins. Such mutant proteins may also lose expression of the conformationally sensitive epitope recognised by the PAb 246 monoclonal antibody and instead express the pan-species conformationally resistant epitope recognised by the PAb 240 antibody (Lane and Benchimol, 1990).

The p53 gene spans 20 kb of DNA on the short arm of human chromosome 17 and has one non-coding exon located several kilobases away from the ten coding exons (Levine, 1990). There are five blocks of evolutionarily conserved amino acid sequence - the majority of mutations alter conserved amino acids in four of these regions (Lane and Benchimol, 1990), and much research has therefore concentrated on the exons coding for these regions: 5, 6, 7 and 8.

Recently, an immense amount of work has been done to characterise p53 mutations in relation to human neoplasia. In lung cancer p53 has been found to be frequently mutated (Takahashi *et al*., 1989) and is expressed abnormally (Iggo *et al*., 1990). Mutation and abnormal expression has also been shown in breast cancer (Coles *et al*., 1990; Thompson *et al*., 1990), gastric carcinoma (Seruca *et al*., 1992), hepatocellular carcinoma (Bressac *et al*., 1990) and many other tumour types (Nigro *et al*., 1989).

Abnormal p53 function has been implicated in haematological malignancies. Mice homozygous for a large deletion within the p53 gene all develop tumours by the age of 6 months, with a high incidence of T-cell lymphomas (Purdie *et al*., 1994). Rearrangement of the gene associated with abnormal p53 expression has been found in chronic myeloid leukaemia (Ahuja *et al*., 1989; Mashal *et al*., 1990). In Burkitt's lymphoma and its leukaemic counterpart mutations of p53 may be associated with an activated c-*myc* oncogene (Gaidano *et al*., 1991) and mutant p53 protein (as detected by the monoclonal antibody PAb 240) is expressed in Burkitt's lymphoma cell lines (Wiman *et al*., 1991). Binding of p53 to human papillomavirus (HPV) E6 protein induces rapid degradation of p53. The presence of wild-type p53 in HPV-positive tumours may indicate that E6 protein binding obviates the need for p53 mutations in the genesis of such tumours, but this association has not been noted with

Correspondence: DJA Adamson, Department of Clinical Oncology, Western General Hospital, Crewe Road, Edinburgh EH4 2XU, UK.
Received 26 August 1994; revised 23 January 1995; accepted 14 February 1995

Epstein–Barr virus (EBV) infection and Hodgkin's disease (Niedobitek et al., 1993). EBV immediate-early protein, BZLF1, mediates lytic replication but can also interact with p53. Immunosuppressed patients have a high frequency of EBV-associated lymphomas, and more than half of these lymphomas have cells which express BZLF1 protein. Inactivation of p53 may unmask viral latency by preventing interaction with this protein, but equally p53 function may be inhibited by an excess of BZLF1 (Zhang et al., 1994). Another EBV protein, EBNA-5 (EBNA-LP), is required for B-cell transformation and can form complexes with both wild-type and mutant p53 (Szekely et al., 1993).

When populations of haemopoietic cells are examined, p53 expression tends to decrease with increasing maturity of the population (Kastan et al., 1991). The progenitor cells as characterised by CD34 and glycophorin positivity have undetectable levels of p53 protein. In contrast, the non-proliferating, mature cells have low but detectable levels of the protein. Immortalised leukaemia cell lines express p53 in a lineage specific manner, with lymphoid cell lines overexpressing and myeloid ones tending to lack expression.

Point mutation is associated with the detection of abnormal p53 expression, but abnormality stabilised protein can exist in the absence of p53 mutation (Wynford-Thomas, 1992). It is also not clear whether immunocytochemistry or mutation analysis indicates the aggressiveness of a lymphoma. We have examined p53 with both mutation analysis (direct sequencing) and with immunocytochemistry using a polyclonal antibody (CM-1) in 38 lymphomas. CM-1 is raised against the wild-type p53 protein (Midgley et al., 1992) but is useful for detecting abnormally stabilised protein as the short half-life of normal p53 protein makes it hard to detect.

### Materials and methods

*Cases*

Approval for the study was obtained from the local Joint Ethics Committee. Tumour samples of Hodgkin's disease and non-Hodgkin's lymphoma (NHL) were collected both fresh from surgical theatres and from samples received in the Department of Pathology, Aberdeen Royal Infirmary, from 1987 onwards and stored at −70°C. Most 'tumour' DNA was extracted from frozen specimens, although some was extracted from fresh samples from theatre before freezing. All the pathological specimens (H&E and CM1 stained) were processed in the same way. The area of the specimen thought most likely to be representative of the tumour was chosen for these analyses, e.g. areas at the periphery of lymph nodes which may have contained normal tissue were avoided. The sections stained with CM1 polyclonal antibody were taken from tumour immediately adjacent to that from which DNA was extracted for SSCP analysis. This could not be guaranteed for every H&E-stained section as some of these were taken from the pathology archives. Cases were selected on the basis that either fresh or archival frozen material was available to provide sections for CM1 staining and also for DNA extraction. The tumours were classified according to the Kiel system (Lennert et al., 1983) and the Working Formulation (Anonymous, 1982). The diagnoses of all tumours were reviewed by one pathologist (WDT) who also scored all the sections stained with CM1.

*DNA extraction*

DNA was extracted from tumour tissue using standard cell lysis and phenol–chloroform purification techniques. The specimens were stored at −20°C if the DNA was not extracted immediately. Genomic DNA stock was kept physically separated from the area where the PCR reactions were prepared.

*Single-strand conformation polymorphism (SSCP) analysis*

Point mutations were sought in exons 5–8 of p53 using 25 ng of template DNA in the amplification reactions and 10 pmol of the following primer pairs:

exon 5 5′-TACTCCCCTGCCCTCAAC-3′
5′-GCCCCAGCTGCTCACCATCG-3′

exon 6 5′-GGCCTCTGATTCCTCACTGATT-3′
5′-AGAGACCCCAGTTGCAA-3′

exon 7 5′-CTTGCCACAGGTCTCCCCAA-3′
5′-AGGGGTCAGCGGCAAGCAGA-3′

exon 8 5′-TGCTTCTCTTTTCCTATCCTGA-3′

5′-CGCTTCTTGTCCTGCTTGCT-3′

The upstream primer of exon 5 was wholly exonic and the downstream primer included the last five bases of the exon. The other primers were wholly intronic. A 'master mix' was used to increase accuracy: 10 × polymerase chain reaction (PCR) buffer [Boehringer Mannheim, Lewis, UK: 100 mmol $l^{-1}$ Tris–HCl, 15–mmol $l^{-1}$ magnesium chloride, 500 mmol $l^{-1}$ potassium chloride, 1 mg $ml^{-1}$ gelatine, pH 8.3 (20°C)]; 'low C' nucleotide mix (nucleotides from Perkin Elmer Cetus, Norwalk, CT, USA) using 10 mmol of dATP, 10 mmol of dGTP, 10 mmol of dTTP, 0.2 mmol of dCTP and sterile water in a ratio of 1:1:1:1:4; 2 µl of a 1:12 dilution of [$\alpha^{32}P$]dCTP (Amersham International, Buckinghamshire, UK); and 0.5 units *Taq* DNA polymerase (Boehringer); overlaid with one drop of mineral oil (Sigma, Poole, UK). The PCR programme was 27 cycles of 94°C for 30 s, 55°C for 30 s and 72°C for 1 min, incorporating [$\alpha^{32}P$]dCTP (Amersham). Point mutations were detected by running the amplification product of a given exon on 8% non-denaturing acrylamide gels with 10% glycerol at 24°C for exon 5 or 5% non-denaturing '2% C' (Hayashi, 1991) acrylamide gels with 5% glycerol at 20°C for exons 6–8. A negative (no template) control was 'amplified' and run to detect PCR contamination and positive controls (samples with known mobility shifts) were run on each gel. Electrophoresis was done on the LKB 2010 Macrophor Sequencing System (LKB-Produkter, Bromma, Sweden) using a water-cooled thermostatic plate and the gels were then soaked in tap water for 15 min to prevent them adhering to the photographic film. After drying the gels were exposed at room temperature to Fuji RX film (Fuji Photo Film, Nomiya, Japan) to obtain a suitable autoradiograph (3–24 h).

*Direct sequencing*

Five pmol of each of the following pairs of primers were used:

exons 5 and 6 5′-TGTTCACTTGTGCCCTGACT-3′
5′-GGAGGGCCACTGACAACCA-3′

exon 7 5′-CAGGTCTCCCCAAGGCGCACTGGCC-3′
5′-TGTGCAGGGTGGCAAGTGGC-3′

exon 8 5′-TGGGAGTAGATGGAGCCTGG-3′
5′-AGGAAAGAGGCAAGGAAAGG-3′

The PCR programme was 30 cycles of 94°C for 2 min, 60°C for 2 min and 72°C for 3 min. The antisense primer was biotinylated (exon 6, King's College, London; exons 5, 7 and 8, Genosys Biotechnologies, Cambridge, UK) at the 5′ end to enable purification of the single-stranded template using streptavidin-conjugated magnetic beads (Dynabeads M-280 Streptavidin, Dynal, Wirral, UK). The sequencing reactions were done with a $^{T7}$Sequencing Kit (Pharmacia Biosystems, Central Milton Keynes, UK) using [$\alpha^{35}S$]dATP (Amersham) and the sense primer for each exon (exon 6 was sequenced using an additional internal sense primer 5′-TGGTTGCCCAGGGTCCCCAG-3′). The DNA fragments generated were separated by electrophoresis on 5% denaturing acrylamide wedge gels. All polyacrylamide gel electrophoresis was done with the LKB 2010 Macrophor Sequencing System (LKB-Produkter) and autoradiography was done by standard methods using Fuji RX photographic film (Fuji Photo Film, Nomiya, Japan).

*Immunocytochemistry with the p53 antibody*

Ovarian tumours known to have abnormal p53 expression and previously investigated with CM1 antibody under the same conditions were used as the positive controls with each batch of lymphoma sections. Negative controls with (a) no CM1 antibody and (b) ovarian samples known to be negative were also prepared. In the samples of Hodgkin's disease the malignant component of the tissue (Reed–Sternberg cells or variants) was used to score the p53 expression. Formalin-fixed, paraffin-embedded lymphoma sections of 5 μm were prepared by the Department of Pathology, Aberdeen Royal Infirmary. The sections were left to dry onto 0.1% poly-L-lysine (Sigma, St Louis, MO, USA)-coated slides overnight at room temperature. The sections were dewaxed in xylene and dehydrated in 100% alcohol. Endogenous peroxidase activity was blocked by methanol–hydrogen peroxide. The section was then preincubated with 20% normal swine serum (Dako, *A/S*, Glostrup, Denmark). CM1 polyclonal antibody (a gift from Dr DP Lane, Department Biochemistry, University of Dundee, UK), used as a 1:1000 dilution, was incubated overnight. The avidin–biotin–peroxidase complex method was used (Hsu *et al.*, 1981). After development with diaminobenzidine the sections were counterstained with haematoxylin, dehydrated and mounted.

### Results

*p53 mutations*

All types of lymphoma were analysed for mutations in exons 5–8. Thirty-three cases of NHL (nine high-grade, 24 low-grade) and five cases of Hodgkin's disease were studied. SSCP analysis was used to screen the 38 samples for single base changes, detected because of the different migration patterns of the wild-type and mutant radiolabelled DNA products when denatured and separated by polyacrylamide gel electrophoresis under non-denaturing conditions. This screening method is fast, specific and sensitive (Gaidano *et al.*, 1991). To confirm and identify the abnormal nature of the DNA products of four samples showing band mobility shifts, direct sequencing was done and showed one silent and three missense mutations. Figure 1 shoes typical abnormal band mobility shifts in the SSCP analysis and Figure 2 shows the silent mutation in a case of Hodgkin's disease. Table I shows the results of the immunocytochemistry by disease subtype. Table II details the four cases where a point mutation was characterised and shows the degree of CM1 antibody positivity for each case.

**Figure 1** SSCP analysis, exon 5, p53. +, positive control. *Cases with a mobility shift.

Three missense mutations were characterised (Table II) in different subtypes of B-cell NHL (one high-grade and two low-grade). The silent mutation (Figure 2) was a case of lymphocyte-depleted Hodgkin's disease, which was predictably CM1 negative. Case 22 (B-lymphoplasmacytoid NHL) was negative for CM1 staining, although the mutation caused glycine to be substituted for the wild-type cysteine at codon 135. This tumour sample also showed allele loss at the YNZ22 locus 20 cM telomeric to p53 (data not shown) when examined by Southern analysis. It was uninformative for markers close to p53 (BHP53 and MCT35.1 – data not shown). The region of chromosome 17p defined by YNZ22 has been implicated with the control of p53 expression (Coles *et al.*, 1990).

**Table I** p53 protein expression measured by CM1 polyclonal antibody staining, with breakdown of lymphoma subtypes (subtypes not examined are omitted from the table)

| *Histological type (Kiel classification)* | *Proportion of positive cases/ all cases* | *Quantification of the p53 positivity* |
|---|---|---|
| *Non-Hodgkin's lymphomas* | | |
| Low-grade B cell | | |
| B-CLL | 1/4 | ++ |
| B-lymphoplasmacytoid | 0/3 | |
| Centroblastic-centrocytic (including B-follicular) | 1/14 | ++ |
| *Low-grade T-cell* | | |
| Angioimmunoblastic lymphadenopathy | 0/2 | |
| All other types | 1/1 | + |
| *High-grade B-cell* | | |
| B-centroblastic diffuse | 3/5 | +/++/++ |
| B-immunoblastic | 2/2 | +/+++ |
| High-grade T cell | | |
| all types | 0/2 | |
| *Hodgkin's disease* | | |
| Lymphocyte predominance | 0/1 | |
| Nodular sclerosis | 1/1 | + |
| Mixed cellularity | 0/2 | |
| Lymphocyte depletion | 0/1 | |

+, <5% of tumour cells positive for CM1 staining; ++, >5% of tumour cells positive for CM1 staining; +++, >>5% of tumour cells positive for CM1 staining.

**Figure 2** Direct sequencing of sample 39 (Hodgkin's disease, lymphocyte depleted) showing a silent mutation in the third last codon of exon 6 of p53.

**Table II** Summary of lymphoma point mutation analysis and immunocytochemistry

| *Case reference* | *Sex/age* | *Lymphoma type* | *Codon* | *Exon* | *Nucleotide* | *Amino acid* | *Clinical stage* | *CMI positivity* |
|---|---|---|---|---|---|---|---|---|
| 39 | M/51 | Hodgkin's disease (lymphocyte depleted) | 222 | 6 | CCG→CCT | Silent (Pro→Pro) | IIB | 0 |
| 22 | M/44 | B-Lymphoplasmacytoid | 135 | 5 | TGC→GGC | Cys→Gly | IV | 0 |
| 27 | F/49 | B-CLL | 133 | 5 | ATG→ATT | Met→Ile | I | >5% (++) |
| 47 | M/56 | B-immunoblastic – high-grade | 258 | 7 | GAA→AAA | Glu→Lys | IV | >>5% (+++) |

*Immunocytochemistry*

CM1 positivity, suggesting abnormal expression or stabilisation of p53 protein, occurred in 8/33 (24%) cases of NHL and one case of Hodgkin's disease ($n = 5$). CM1 staining was positive in seven cases in which no point mutation was detected.

### Discussion

Burkitt's lymphoma has been quite extensively analysed for point mutation of p53 (Gaidano *et al.*, 1991; Bhatia *et al.*, 1992), but other lymphomas have not been so well characterised, although one study (Ichikawa *et al.*, 1992) of point mutation in exons 5–8 in B-cell lymphoma identified mutation in 9/48 patients. Eight of these patients had advanced disease (stage IV). Kocialkowski *et al.* (1995) have found mutations in exons 4 and 10 (3/10 positive cases out of 22 cases of high grade NHL). Abnormalities of p53 have been linked to progression in follicular lymphomas (Lo Coco *et al.*, 1993) and one study found that a majority of tumours with point mutation also had abnormal p53 expression (Sander *et al.*, 1993). Wada *et al.* (1993) found that only 2% (8/330) of childhood lymphoid malignancies had mutated p53 (exons 5–8), but 2/8 of these mutations were in B-cell NHL. Abnormally expressed p53 has been detected in Reed–Sternberg cells in Hodgkin's disease (Gupta *et al.*, 1992) and has been found to be associated with point mutation (Trümper *et al.*, 1993). The latter group used single-cell analysis, which avoids the sensitivity problems caused by the large number of non-malignant cells in this disease.

There are several reasons why abnormal immunocytochemistry may not always correlate with point mutation of p53. Immunocytochemical negative results might be caused by gross deletion of the p53 gene, leading to abolition of protein production, but this is likely to be rare, and even a mutation inserting a stop codon would probably cause production of enough p53 protein to be detectable by CM1. Splice site mutation, such as that reported in chronic myeloid leukaemia (Foti *et al.*, 1990), might lead to a decreased availability of epitopes for the antibody to bind to, giving a negative result in the presence of a mutation. Apart from technical artifact, another possibility is that the point mutation does not stabilise the protein sufficiently to raise its concentration to a level that can be detected by CM1 immunocytochemistry. Evidence for this argument is provided by experiments which show that the strong uniform staining of thyroid cancer cell lines becomes weaker or undetectable when such cell lines are grown as tumours in immunodeficient hosts. The positive staining returns when the cells are regrown in culture (Wynford-Thomas, 1992). In addition, a non-mutational mechanism for p53 stabilisation has been proposed for those benign tissues which occasionally have positive staining for p53 (Villuendas *et al.*, 1992).

Seven out of nine of those tumours staining positive showed no point mutation in exons 5–8 of p53, and one of the three lymphomas with missense mutations showed no positivity with CM1 immunocytochemistry (sample 22). Lymphoma may be a disease which has an atypical spectrum of mutations outwith the exons usually involved in neoplasia, such as exons 4 and 9, which were not analysed for point mutation in this study. Kocialkowski *et al.* (1995) have demonstrated that this is the case in at least some cases of high-grade NHL. In addition, it is possible that the design of our exon 5 primers allowed some point mutations to remain undetected. Alternatively, positive immunocytochemistry apparently without point mutation could be attributed to insensitivity of the SSCP analysis or the direct sequencing of mutated exons when the presence of normal DNA masks the abnormality. Against this, however, is the finding of positive immunocytochemistry in *clonal* cell lines which have no p53 mutation – in addition, p53 may be stabilised by *ras* and thereby cause positive immunocytochemistry without gene mutation (Wynford-Thomas, 1992). If such stabilisation of p53 protein occurs regularly *in vivo* without mutation of the gene then immunocytochemistry may provide more information about the neoplastic potential of a tumour than mutation analysis would.

Positive p53 immunocytochemistry may be associated with more aggressive neoplasia in lymphoma (Villuendas *et al.*, 1992) and prostatic carcinoma (Visakorpi *et al.*, 1992). However, we would agree with other groups (Nakamura *et al.*, 1993; Kocialkowski *et al.*, 1995) that, despite the association between p53 expression and mutation in certain tumours, immunocytochemistry cannot be used to determine which tumours have mutations of p53. Soini *et al.* (1992) found that the majority of their tumours which had abnormal p53 expression were of high-grade type. Although the use of archival material may have influenced the selection of tumours studied, it is interesting that in this study 5/9 'high-grade' NHL tumours but only 3/24 'low-grade' NHL tumours stained positive for p53.

**Acknowledgements**

This work was supported by the Milne Bequest to the University of Aberdeen and by Grampian Health Board.

### References

AHUJA H, BAR-ELI M, ADVANI SH, BENCHIMOL S AND CLINE MJ. (1989). Alterations in the p53 gene and the clonal evolution of the blast crisis of chronic myelocytic leukemia. *Proc. Natl Acad. Sci. USA*, **86**, 6783–6787.

ANONYMOUS. (1982). National Cancer Institute sponsored study of classification of non-Hodgkin's lymphomas: summary and description of a working formulation for clinical usage. The non-Hodgkin's Lymphoma Pathologic Classification Project. *Cancer*, **49**, 2112–2135.

BHATIA KG, GUTIERREZ MI, HUPPI K, SIWARSKI D AND MAGRATH IT. (1992). The pattern of p53 mutations in Burkitt's lymphoma differs from that of solid tumors. *Cancer Res.*, **52**, 4273–4276.

BRESSAC B, GALVIN KM, LIANG TJ, ISSELBACHER KJ, WANDS JR AND OZTURK M. (1990). Abnormal structure and expression of p53 gene in human hepatocellular carcinoma. *Proc. Natl Adac. Sci. USA*, **87**, 1973–1977.

CLARKE AR. PURDIE CA. HARRISON DJ. MORRIS RG. BIRD CC. HOOPER ML AND WYLLIE AH. (1993). Thymocyte apoptosis induced by p53-dependent and independent pathways. *Nature*, **362**, 849-852.

COLES C. THOMPSON AM. ELDER PA. COHEN BB. MACKENZIE IM. CRANSTON G. CHETTY U. MACKAY J. MACDONALD M. NAKAMURA Y. HOYHEIM B AND STEEL CM. (1990). Evidence implicating at least two genes on chromosome 17p in breast carcinogenesis. *Lancet*, **336**, 761-763.

EL-DEIRY WS. KERN SE. PIETENPOL JA. KINZLER KW AND VOGELSTEIN B. (1992). Definition of a consensus binding site for p53. *Nature Genet.*, **1**, 45-49.

FOTI A. BAR-ELI M. AHUJA HG AND CLINE MJ. (1990). A splicing mutation accounts for the lack of p53 gene expression in a CML blast crisis cell line: a novel mechanism of p53 gene inactivation. *Br. J. Haematol.*, **76**, 143-145.

GAIDANO G. BALLERINI P. GONG JZ. INGHIRAMI G. NERI A. NEWCOMB EW. MAGRATH IT. KNOWLES DM AND DALLAFAVERA R. (1991). P53 mutations in human lymphoid malignancies association with Burkitt lymphoma and chronic lymphocytic leukemia. *Proc. Natl Acad. Sci. USA*, **88**, 5413-5417.

GUPTA RK. NORTON AJ. THOMPSON IW. LISTER TA AND BODMER JG. (1992). p53 expression in Reed-Sternberg cells of Hodgkin's disease. *Br. J. Cancer*, **66**, 649-652.

HARTWELL L. (1992). Defects in a cell cycle checkpoint may be responsible for the genomic instability of cancer cells. *Cell*, **71**, 543-546.

HAYASHI K. (1991). PCR-SSCP: a simple and sensitive method for detection of mutations in the genomic DNA. *PCR Methods Appl.* **1**, 34-38.

HOLLSTEIN M. SIDRANSKY D. VOGELSTEIN B AND HARRIS CC. (1991). p53 mutations in human cancers. *Science*, **253**, 49-53.

HSU SM. RAINE L AND FANGER H. (1981). A comparative study of the peroxidase-antiperoxidase method and an avidin-biotin complex method for studying polypeptide hormones with radioimmunoassay antibodies. *Am. J. Clin. Pathol.*, **75**, 734-738.

ICHIKAWA A. HOTTA T. TAKAGI N. TSUSHITA K. KINOSHITA T. NAGAI H. MURAKAMI Y. HAYASHI K AND SAITO H. (1992). Mutations of p53 gene and their relation to disease progression in B-cell lymphoma. *Blood*, **79**, 2701-2707.

IGGO R. GATTER K. BARTEK J. LANE D AND HARRIS AL. (1990). Increased expression of mutant forms of p53 oncogene in primary lung cancer. *Lancet*, **335**, 675-679.

KASTAN MB. RADIN AI. KUERBITZ SJ. ONYEKWERE O. WOLKOW CA. CIVIN CI. STONE KD. WOO T. RAVINDRANATH Y AND CRAIG RW. (1991). Levels of p53 protein increase with maturation in human haemopoietic cells. *Cancer Res.*, **51**, 4279-4286.

KOCIALKOWSKI S. PEZZELLA F. MORRISON H. JONES M. LAHA S. HARRIS AL. MASON DY AND GATTER KC. (1995). Mutations in the p53 gene are not limited to classic 'hot spots' and are not predictive of p53 protein expression in high-grade non-Hodgkin's lymphoma. *Br. J. Haematol.*, **89**, 55-60.

LANE DP. (1992). p53, guardian of the genome. *Nature*, **358**, 15-16.

LANE DP. (1993)· A death in the life of p53. *Nature*, **362**, 786-787.

LANE DP AND BENCHIMOL S. (1990). p53: oncogene or antioncogene? *Genes Dev.*, **4**, 1-8.

LENNERT K. COLLINS RD AND LUKES RJ. (1983). Concordance of the Kiel and Lukes-Collins classifications of non-Hodgkin's lymphomas. *Histopathology*, **7**, 549-559.

LEVINE AJ. (1990). The p53 protein and its interactions with the oncogene products of the small DNA tumor viruses. *Virology*, **177**, 419-426.

LEVINE AJ. (1995). Tumour suppressor genes. *Sci. Am. Sci. Med.*, **2**, 28-37.

LEVINE AJ. MOMAND J AND FINLAY CA. (1991). The p53 tumour suppressor gene. *Nature*, **351**, 453-456.

LIN D. SHIELDS MT. ULLRICH SJ. APPELLA E AND MERCER WE. (1992). Growth arrest induced by wild-type p53 protein blocks cells prior to or near the restriction point in late $G_1$ phase. *Proc. Natl Acad. Sci. USA.*, **89**, 9210-9214.

LO COCO F. GAIDANO G. LOUIE DC. OFFIT K. CHAGANTI RSK AND DALLA-FAVERA R. (1993). p53 mutations are associated with histological transformation of follicular lymphoma. *Blood*, **82**, 2289-2295.

MARX J. (1989). Many gene changes found in cancer. *Science*, **246**, 1386-1388.

MASHAL R. SHTALRID M. TALPAZ M. KANTARJIAN H. SMITH L. BERAN M. CORK A. TRUJILLO J. GUTTERMAN J AND DEISSEROTH A. (1990). Rearrangement and expression of p53 in the chronic phase and blast crisis of chronic myelogenous leukaemia. *Blood*, **75**, 180-189.

MIDGLEY CA. FISHER CJ. BÁRTEK J. VOJTĚŠEK B. LANE D AND BARNES DM. (1992). Analysis of p53 expression in human tumours: an antibody raised against human p53 expressed in *Escherichia coli*. *J. Cell. Sci.*, **101**, 183-189.

MOMAND J. ZAMBETTI GP. OLSON DC. GEORGE D AND LEVINE AJ. (1992). The *mdm*-2 oncogene product forms a complex with the p53 protein and inhibits p53-mediated transactivation. *Cell*, **69**, 1237-1245.

NAKAMURA H. SAID JW. MILLER CW AND KOEFFLER HP. (1993). Mutation and protein expression of p53 in acquired immunodeficiency syndrome-related lymphomas. *Blood*, **82**, 920-926.

NIGRO JM. BAKER SJ. PREISINGER AC. JESSUP JM. HOSTETTER R. CLEARY K. BIGNER SH. DAVIDSON N. BAYLIN S. DEVILEE P. GLOVER T. COLLINS FS. WESTON A. MODALI R. HARRIS CC AND VOGELSTEIN B. (1989). Mutations in the p53 gene occur in diverse tumour types. *Nature*, **342**, 705-708.

NIEDOBITEK G. ROWLANDS DC. YOUNG LS. HERBST H. WILLIAMS A. HALL P. PADFIELD J. ROONEY N AND JONES EL. (1993). Overexpression of p53 in Hodgkin's disease: lack of correlation with Epstein-Barr virus infection. *J. Pathol.*, **169**, 207-212.

PIETENPOL JA AND VOGELSTEIN B. (1993). No room at the p53 inn. *Nature*, **365**, 17-18.

PURDIE CA. HARRISON DJ. PETER A. DOBBIE L. WHITE S. HOWIE SEM. SALTER DM. BIRD CC. WYLLIE AH. HOOPER ML AND CLARKE AR. (1994). Tumour incidence, spectrum and ploidy in mice with a large deletion in the p53 gene. *Oncogene*, **9**, 603-609.

SANDER CA. YANO T. CLARK HM. HARRIS C. LONGO DL. JAFFE ES AND RAFFELD M. (1993). p53 mutation is associated with progression in follicular lymphomas. *Blood*, **82**, 1994-2004.

SERUCA R. DAVID L. HOLM R. NESLAND JM. FANGAN BM. CASTEDO S. SOBRINHO-SIMÕES M AND BØRRESEN A-L. (1992). p53 mutations in gastric carcinomas. *Br. J. Cancer*, **65**, 708-710.

SOINI Y. PÄÄKKÖ P. ALAVAIKKO M AND VÄHÄKANGAS K. (1992). p53 expression in lymphatic malignancies. *J. Clin. Pathol.*, **45**, 1011-1014.

SZEKELY L. SELIVANOVA G. MAGNUSSON KP. KLEIN G AND WIMAN KG. (1993). EBNA-5, an Epstein-Barr virus-encoded nuclear antigen, binds to the retinoblastoma and p53 proteins. *Proc. Natl Acad. Sci. USA*, **90**, 5455-5459.

TAKAHASHI T. NAU MM. CHIBA I. BIRRER MJ. ROSENBERG RK. VINOCOUR M. LEVITT M. PASS H. GAZDAR AF AND MINNA JD. (1989). p53: a frequent target for genetic abnormalities in lung cancer. *Science*, **246**, 491-494.

THOMPSON AM. STEEL CM. CHETTY U. HAWKINS RA. MILLER WR. CARTER DC. FORREST APM AND EVANS HJ. (1990). p53 gene mRNA expression and chromosome 17p allele loss in breast cancer. *Br. J. Cancer*, **61**, 74-78.

TRÜMPER LH. BRADY G. BAGG A. GRAY D. LOKE SL. GRIESSER H. WAGMAN R. BRAZIEL R. GASCOYNE RD. VICINI S. ISCOVE NN. COSSMAN J AND MAK TW. (1993). Single-cell analysis of Hodgkin and Reed-Sternberg cells: molecular heterogeneity of gene expression and p53 mutations. *Blood*, **81**, 3097-3115.

VILLUENDAS R. PIRIS MA. ORRADRE JL. MOLLEJO M. ALGARA P. SANCHEZ L. MARTINEZ JC AND MARTINEZ P. (1992). p53 protein expression in lymphomas and reactive lymphoid tissue. *J. Pathol.*, **166**, 235-241.

VISAKORPI T. KALLIONIEMI O-P. HEIKKINEN A. KOIVULA T AND ISOLA J. (1992). Small subgroup of aggressive, proliferative prostatic carcinomas defined by p53 accumulation. *J. Natl Cancer Inst.*, **84**, 883-887.

WADA M, BARTRAM CR. NAKAMURA H. HACHIYA M. CHEN D-L. BORENSTEIN J. MILLER CW. LUDWIG L. HANSEN-HAGGE TE. LUDWIG W-D. REITER A. MIZOGUCHI H AND KOEFFLER HP. (1993). Analysis of p53 mutations in a large series of lymphoid hematologic malignancies of childhood. *Blood*, **82**, 3163-3169.

WIMAN KG. MAGNUSSON KP. RAMQVIST T AND KLEIN G. (1991). Mutant p53 detected in a majority of Burkitt lymphoma cell lines by monoclonal antibody PAb240. *Oncogene*, **6**, 1633-1639.

WYNFORD-THOMAS D. (1992). p53 in tumour pathology: can we trust immunocytochemistry? *J. Pathol.*, **166**, 329-330.

ZHANG Q. GUTSCH D AND KENNEY S. (1994). Functional and physical interaction between p53 and BZLF1: implications for Epstein-Barr virus latency. *Mol. Cell. Biol.*, **14**, 1929-1938.